# Establishment and characterization of HXWMF-1: the first mouse fibroblastic tumor cell line derived from leukemia-associated fibroblasts

**DOI:** 10.1186/s12935-021-01870-7

**Published:** 2021-03-19

**Authors:** Yuanyuan Li, Ling Gu

**Affiliations:** 1 Laboratory of Hematology/Oncology, Department of Pediatrics, Key Laboratory of Birth Defects and Related Diseases of Women and Children (Sichuan University), Ministry of Education, West China Second University Hospital, Sichuan University, No. 20, Section 3, Renmin South Road, Chengdu, 610041 People’s Republic of China; 2 Joint Laboratory of West China Second University Hospital, Sichuan University and School of Life Science, Fudan University for Pulmonary Development and Disease, Chengdu, 610041 China

**Keywords:** Cancer-associated fibroblasts, Acute lymphoblastic leukemia, Leukemic environment, Cell line

## Abstract

**Background:**

Chemo-resistance is still a major obstacle in leukemia treatment. Accumulating evidence indicates that cancer-associated fibroblasts (CAFs), the most abundant stromal cells in tumor microenvironment (TME), play a crucial role in cancer progression and response to chemotherapy. To Figure out the role of leukemia-associated fibroblasts (LAFs) in relapsed/refractory leukemia, we constructed the first leukemia-associated fibroblastic tumor cell line, HXWMF-1.

**Methods:**

A cell culture technique was used to establish the leukemia-associated fibroblastic tumor cell line. Molecular and cellular biological techniques including flow cytometry, MTT assay, western blotting, and short tandem repeat (STR) analysis were used to characterize the cell line. Nude mice were used for xenograft studies.

**Results:**

We established a LAFs derived tumor cell line HXWMF-1, originated from the subcutaneous xenografts of HXEX-ALL1, a cell line originated from a patient with acute lymphoblastic leukemia (ALL) at the second relapse. The HXWMF-1 cell line was authenticated as a tumor cell line and being derived from CAFs based on morphologic, immunophenotypic, cytogenetic and STR analyses and tumorigenicity test in nude mice. To clarify the reliability of the method, we got the LAFs derived tumor cells from three different tumor mass of HXEX-ALL1 xenografts.

**Conclusions:**

To our knowledge, HXWMF-1 is the first fibroblastic tumor cell line derived from LAFs or CAFs. In addition, the cell line provided firm evidence for that leukemia cells may induce LAFs/CAFs malignant transformation, which may help to develop brand new theory and therapeutic strategies for patients with relapsed /refractory ALL.

## Background

Despite great progress in curing acute lymphoblastic leukemia (ALL), survival after relapse remains poor [1–3]. Much of the success has been driven by refinements in risk stratification, but unfortunately, intensity of conventional chemotherapy has reach its limit of tolerance and can no longer pushed to obtain improved results [4]. Chemo-resistance remains the main challenge in successful treatment of ALL. Efforts to understand resistance to chemotherapy focused mainly on cell-intrinsic mechanism, and got few clinic benefits. To date, leukemic cells are the only drug-target in almost all the therapeutic protocols. Recently, increasing evidences indicated that cancer progression and metastasis is controlled by the tumor microenvironment (TME) and does not depend solely on cancer cell-autonomous defects, and TME should be a key target for cancer therapy [5–8]. Similarly, leukemic stem cells reside in bone marrow (BM), interact with BM microenvironment (niche) and alter the niche to facilitate their own growth and evade chemotherapy [9–12]. TME is a multicellular system with complex tumor-stromal interactions, and has been addressed five emerging concepts: its dynamic evolution, how it is educated by tumor cells, pathways of communication between stromal and tumor cells, immunomodulatory roles of the lymphatic system, and contribution of the intestinal microbiota [13]. Now, to explore the therapeutic target in TME has become the key point in cancer treatment.

A dominant component of the tumor stroma is fibroblasts, which has been extensively concerned owing to their role in cancer initiation, progression, and metastasis [14, 15]. Fibroblasts associated with cancer have been termed cancer-associated fibroblasts (CAFs), tumor-associated fibroblasts (TAFs), activated fibroblasts or activated myofibroblasts. CAFs are the most abundant stromal cells within TME [14, 15]. Although CAFs have been explored in depth in many solid tumors, only a few studies have directly addressed the role of the CAFs in leukemia. For that, BM biopsies are not routinely performed in patients with leukemia [16]. Recently, papers reported that CAFs are widespread in the BM and play a pivotal role in cancer progress and chemoresistance in hematological malignances, multiple myeloma, chronic lymphoblastic leukemia, acute myeloblastic leukemia (AML), and ALL [16–21]. In addition, excessive reticular fibers in the BM led to a poor prognosis in ALL and AML patients [22, 23], making it important to investigate the functional roles of the fibroblasts.

Activated fibroblasts isolated from various human tumors exhibit many distinct properties when compared with fibroblasts cultured from normal organs [14, 15, 24]. Cell lines established from tumors are the most commonly used models in cancer research, which have promoted a better understanding of the biology of cancer and helped to develop effective treatment strategies [25]. To explore the interaction of CAFs and chemo-resistant leukemic cells, we tried to establish leukemia-associated fibroblasts (LAFs) cell lines from xenografts of HXEX-ALL1, a B-ALL cell line, for that the cell line was established from a 6-years-patient of B-ALL relapsed two times after contemporary risk-directed chemotherapy [26]. Unexpected and surprised, we got a fibroblastic tumor cell line from the xenografts, which might help to understand the crosstalk between TME and cancer cells, especially in patients with refractory or relapsed cancer.

## Methods

### Cell line establishment and culture

HXEX-ALL1, a pediatric B-ALL cell line established in our laboratory [26]**,** was cultured as reported [26]. Cultured 5 × 10^6^ HXEX-ALL1 cells were subcutaneously injected into the right flanks of 6-week-old female BALB/c (nu/nu) nude mice. After 28 days, tumor mass was dissected and minced with scissors. Cells, isolated from the resected tumor mass, were cultured in vitro in RPMI-1640 medium (Gibco) supplemented with 10% FBS (Thermo) at 37 °C with 5% CO_2_. After 3 days, a little part of cells was attached with spindle or long-spindle shape. Then, the medium was changed to remove floating cells. Passage was performed until 80% confluence of cells. After 24 days, cells with long-spindle shape disappeared, and the attached cells became spindle or polygon in shape. After 40 days of lag phase, the cell number dramatically increased, and the cell density was adjusted to 1.5–2 × 10^5^/55 cm^2^. Cells at 80% confluency were split in the ratio of 1:5 or 1:6 every 2–3 days. The cells were examined daily under an inverted microscope, and the cell number was determined every 2 days with a standard hematocytometer using trypan blue dye exclusion. The obtained cells were designated HXWMF-1.

### Cell morphologic assay

Morphological characteristics of live cultured cells were observed under an inverted microscope (Olympus, MA, USA). HXWMF-1 cells of climbing flake were stained with Wright-Giemsa and observed under an optical microscope (Olympus). Cell ultrastructure was observed with scanning electron microscope (SEM) (FEI Company, OR, USA) and transmission electron microscope (TEM) (JEOL Ltd., Tokyo, Japan).

### Cell growth assay

Cells were cultured in a 6-well plastic culture plates (Corning Inc., Corning, NY, USA) at 2 × 10^3^/9.6cm^2^ in RPMI-1640 medium with 10% FBS and grown for eight days. Viable cells were counted using trypan blue (Sigma, St. Louis, MO, UAS) staining every day. Td (doubling time) was calculated for cells in exponential growth with the following equation: Td (h) = t × lg2/lg(N_t_/N_0_), where t is the time of continuous culture, N_t_ is the final number of cells, and N_0_ is the initial number of cells. Cell viability was evaluated by the 3-(4,5 dimethylthiazol-2-yl)-2,5-diphenyltetrazolium bromide (MTT) assay.

### Cell cycle analysis

For each analysis, 10^6^ cells were harvested and fixed overnight in 70% ethanol at 4 °C. The cells were then washed and stained with 5 μg/ml PI in the presence of DNAse-free RNAse (Sigma). After 30 min at room temperature, the cells were analyzed via flow cytometry (Beckman Coulter Inc., Miami, FL, USA) with the acquisition of 30,000 events.

### Cell colony formation assay

Logarithmically growing cells were harvested and seeded in 6-well sterile plastic culture plates at a density of 2 × 10^2^ cells /9.6 cm^2^ in methylcellulose RPMI-1640 medium containing 0.9% methylcellulose (MethoCult GFH4434; Sigma, St. Louis, MO, USA) and 10% FBS at 37 °C under a humidified atmosphere with 5% CO_2_ and 21% O_2_. When the visible colonies appeared in the dish, the culture was terminated. The clones were fixed with 5 mL of 4% paraformaldehyde for 15 min, and then stained with crystal violet for 10–30 min. Imaging and cell counting were performed under an inverted microscope (Olympus, Tokyo, Japan). The clone formation rate (%) = number of clones formed/number of cells inoculated × 100%. Random aspiration of individual colonies growing in methylcellulose was undertaken on day 7 of the culture. Next, each clone was cultured in RPMI-1640 complete medium.

### Short tandem repeat analysis

The identity of the HXWMF-1 cell line was checked using short tandem repeat (STR) analysis. DNA was prepared from whole HXWMF-1 cells using a genomic DNA kit (Microread Genetics, Beijing, China) according to the manufacturer’s instructions. The following 12 highly polymorphic STR loci were tested by multiplex PCR: 18–3, 9–2, 6–7, 5–5, X-1, 15–3, 12–1, 6–4, csf1po, VWA, 4–2 and jarid1.

### Chromosome analysis

In brief, cells were incubated with standard medium containing 100 ng/ml colcemid for 2 h. Chromosomes were prepared by a standard method and analyzed by the G-banding technique.

### Immunophenotypic analysis

For the detection of the immunophenotype of the HXWMF-1 cells, we used antibodies against the following targets: fibroblast specific protein 1 (FSP1, also named as S100A4), CD34, LY-6A/E (Sca-1) and CD166 (Becton Dickinson Inc., Franklin Lakes, NJ, USA). Expression of the markers was determined using a flow cytometry (Beckman Coulter Inc.).

### Western blotting analysis

Western blotting analysis was performed on lysates obtained from HXWMF-1, HXEX-ALL1, and BaF3 (a mouse pro-B cell line) cells. Proteins were separated by 15% SDS-polyacrylamide gel electrophoresis and transferred onto nitrocellulose membranes (0.45 or 0.22 μm, Millipore, Billerica, MA, USA). Proteins were visualized by incubation with ECL plus reagent (Millipore). All experiments were independently carried out at least 3 times. The level of β-actin protein was used as a control for the amount of protein loaded onto each lane.

### In vivo experiments

Cultured 3 × 10^6^ HXWMF-1 cells were subcutaneously injected into the right flanks of 5-week-old female BALB/c (nu/nu) nude mice, with 0.2 ml of PBS injected into the left flanks as the control (*n* = 6). Tumor size was measured by calipers every 2 days. The approximate tumor volume was calculated using the equation V = (length × width × width)/2. All animal care was in compliance with the guidelines established by the internal Institutional Animal Care and Use Committee and Ethics Committee of Sichuan University. After the mice were euthanized, the tumor mass was excised, fixed in 10% formalin, and routinely processed for paraffin embedding. Five-millimeter-thick sections were obtained and prepared for standard histopathological examination.

### Migration assay

A wound healing assay was done to measure the closure of a scratch and the migration rate of the cells. Cells were incubated for 3 days and images were taken on each day under inverted microscopy.

### Statistical analysis

All assays were performed in triplicate, and the data were expressed as the mean value ± SD. One-way ANOVA was used to compare two groups. A *P*-value < 0.05 was considered to be significant.

## Results

### Establishment of the HXWMF-1 cell line

A stable cell population was constructed following 40 days of culture of primary dissected tumor mass. Initially, contamination of long spindle-shaped cells and adipose cells were observed, but they disappeared from the cultures upon passaging the cells. The cells were maintained in fresh medium (RPMI-1640 medium containing 10% FBS) passaged at 2- to 3-days intervals. The obtained cells were designated HXWMF-1 and made available. The HXWMF-1 cells had been in culture continuously for more than 18 months, and passaged more than 180 times, and more than 800 population doubling levels (PDLs) were successively carried out. The cells proliferated consistently and were negative for mycoplasma infection based on PCR. The cells could be frozen under standard conditions using 60% RPMI-1640 medium, 30% FBS and 10% dimethyl sulfoxide (DMSO) and successfully revived after storage in liquid nitrogen, with more than 80% viability. The cells maintained the same properties after freezing and thawing.

### Morphological characteristics of HXWMF-1 cells

**Fig. 1:**
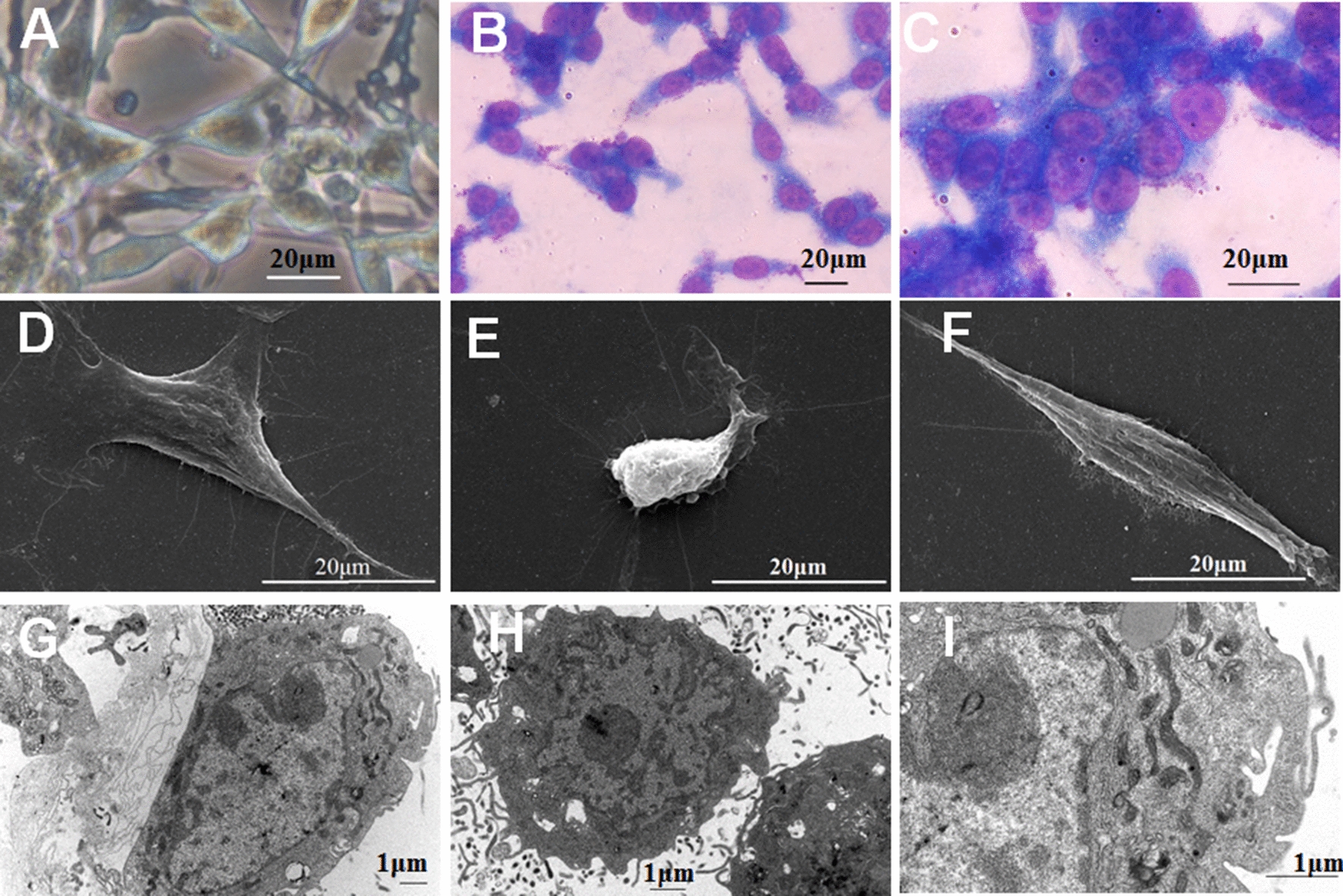
Morphological characteristics of HXWMF-1 cell line. **a** Morphology of HXWMF-1 cells under a light microscopy (×400 magnification). Cells grew mainly in clusters of spindle or polygonal shape. **b**, **c** Wright-Giemsa staining of HXWMF-1 cells (**b** ×200 magnification; **c** ×400 magnification) showed the nucleo-cytoplasmic ratio of these cells was high. **d**–**f** Ultrastructural appearance under a SEM (×5000 magnification) showed spindle or polygonal shaped cells with densely filamentous microvilli and lamellar prominences on the surface. **g**–**i** Ultrastructural appearance under a TEM (**g** ×10,000 magnification, **h** ×12,000 magnification, **i** ×25,000 magnification) showed lots of microvilli outside the network and cell connection and clear cellular organelle

After plating, spindle-shaped HXWMF-1 cells adhered to wall when it was maintained under monolayer. Before reaching confluence, the cells lost their ability of postconfluence contact inhibition of division, and grew in overlap of spindle or polygonal shape (Fig. 1a). Wright-Giemsa stain showed that the cells contained a single nucleus that was commonly ovoid or round, with big nucleoplasm ratio and several prominent nucleoli (Fig. 1b, c). Moreover, spindle or polygonal shaped cells with densely filamentous microvilli and lamellar prominences on the surface, lots of microvilli outside the network and cell connection, and large nucleus with clear cellular organelle structures such as ribosome, mitochondria, endoplasmic reticulum, lysosome, glycogen and secretory granules in cytoplasm, were clearly visualized under a TEM and SEM (Fig. 1d–i).

### Proliferation of HXWMF-1 cells

**Fig. 2:**
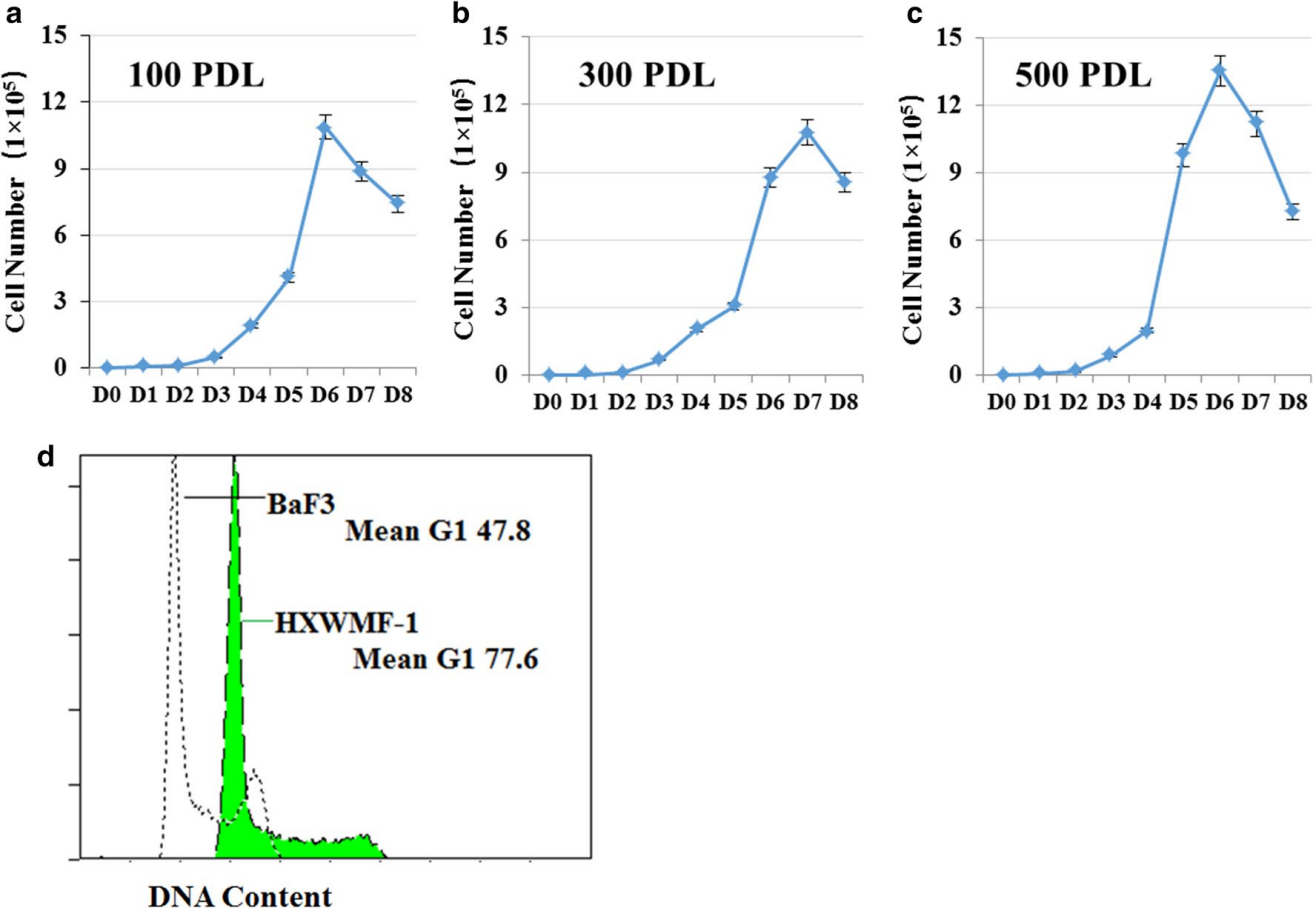
Growth curve of HXWMF-1 cells at different PDLs. Cells were cultured in a 6-well culture plate at 2 × 10^3^/9.6 cm^2^ in RPMI-1640 medium with 10% FBS and grown for 8 days. Viable cells were counted using trypan blue staining every day. Experiments were performed in triplicate. **a** 100 PDLs. **b** 300 PDLs. **c** 500 PDLs. **d** DNA index showed DNA aneuploid of HXWMF-1. Cell cycle was analyzed by PI staining. DNA index was calculated as the ratio of aneuploid to diploid G0/G1 peak channel

We determined the proliferation for 8 days at different cell PDLs of HXWMF-1 cells (Fig. 2a–c). The results revealed that HXWMF-1 cells stably proliferated in RPMI-1640 medium containing 10% FBS with a population Td of 12 to 17 h without significant differences (*P* > 0.05) at different PDLs. Flow cytometry revealed that the DNA index was 1.63 ± 0.05 with an aneuploidy cycle (Fig. 2d). Our results suggesting that the HXWMF-1 cells could stably proliferation during continuous culture in vitro.

### Authorization of HXWMF-1 cells

**Fig. 3:**
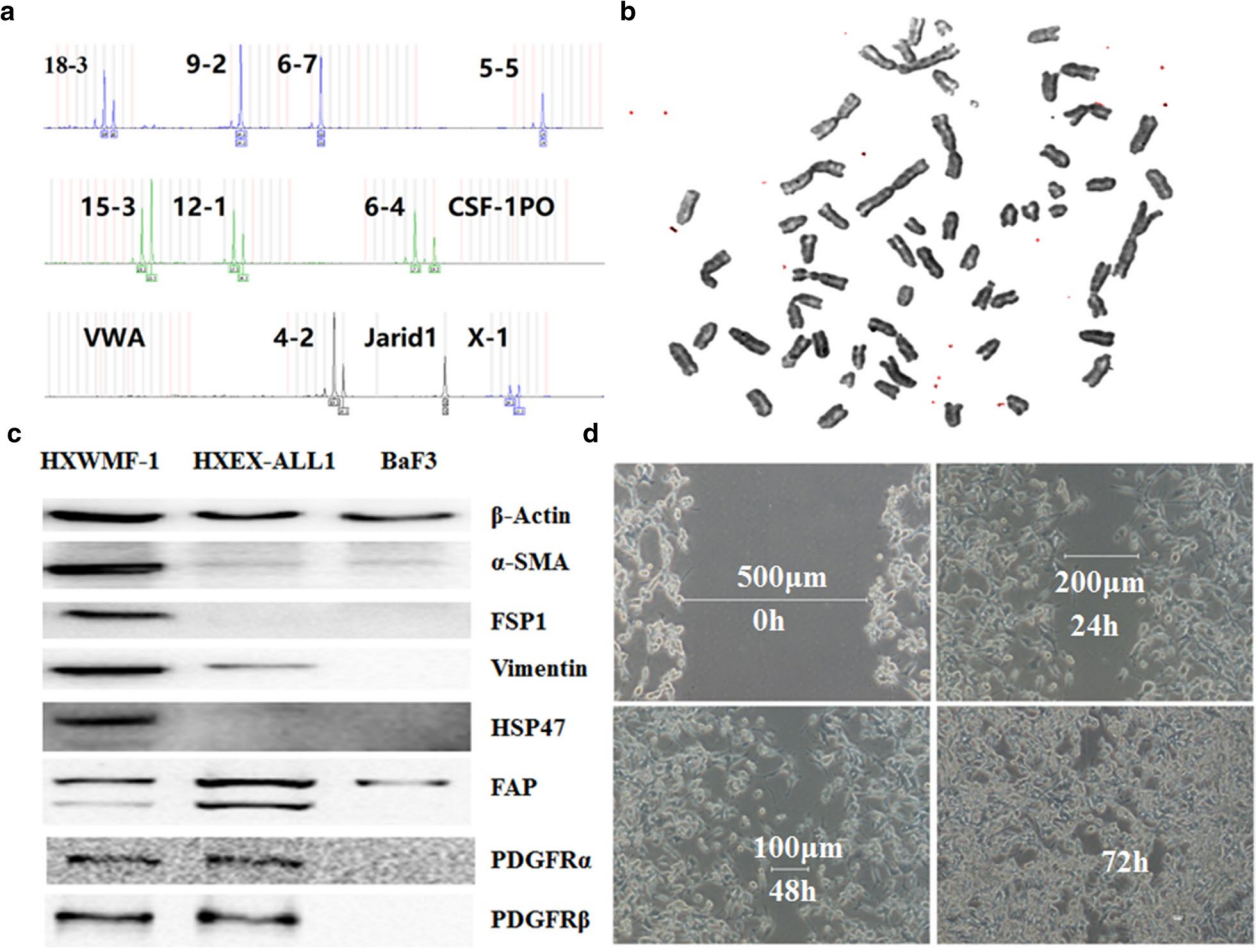
Authorization of HXWMF-1 cells. **a** STR of HXWMF-1 cells showed that HXWMF-1 cells were originated from mouse. **b** G-banding karyotype showed a complex chromosome aberration with total 60–70 chromosomes in HXWMF-1 cells. **c** Fibroblastic cell markers of HXWMF-1 cells. Cells were lysed, and extracts were analyzed by western blotting for α-SMA, FSP1, Vimentin, HSP47, FAP, PDGFRα and PDGFRβ. β-Actin was used as an internal control. Experiments were performed in triplicate. **d** Wound healing assay showed that HXWMF-1 cells closed the scratched area in 72 h

Using a multiplex STR system, we verified that the HXWMF-1 cells were originated from mouse (Fig. 3a, Table 1). G-banding analysis showed that HXWMF-1 cells have 60–70 chromosomes with complex structural chromosomal abnormalities (Fig. 3b). To confirm the expression of fibroblastic cell markers in HXWMF-1 cells, FSP1, CD34, LY-6A/E and CD166 were measured positive by flow cytometry. Western blotting confirmed that α-smooth muscle actin (α-SMA), FSP1, Vimentin, heat shock protein 47 (HSP47), fibroblast activation protein (FAP), and platelet-derived growth factor receptor (PDGFR) were highly expressed in HXWMF-1 cells (Fig. 3c). The migratory ability of HXWMF-1 cells was analyzed with the wound healing assay, and the results showed that HXWMF-1 cells closed the scratched area in 72 h (Fig. 3d).

**Table 1:** STR analysis of HXWMF-1 cells

Markers	Allele 1	Allele 2
18-3	19	20
9-2	14.3	14.3
6-7	12	12
5-5	14	14
X-1	24.3	25.3
15-3	21.3	22.3
12-1	15.3	16.3
6-4	17.3	19.3
CSF1PO	–	–
vWA	–	–
4-2	19.3	20.3
Jarid1	X	X

### The tumorigenicity of HXWMF-1 cells

**Fig. 4:**
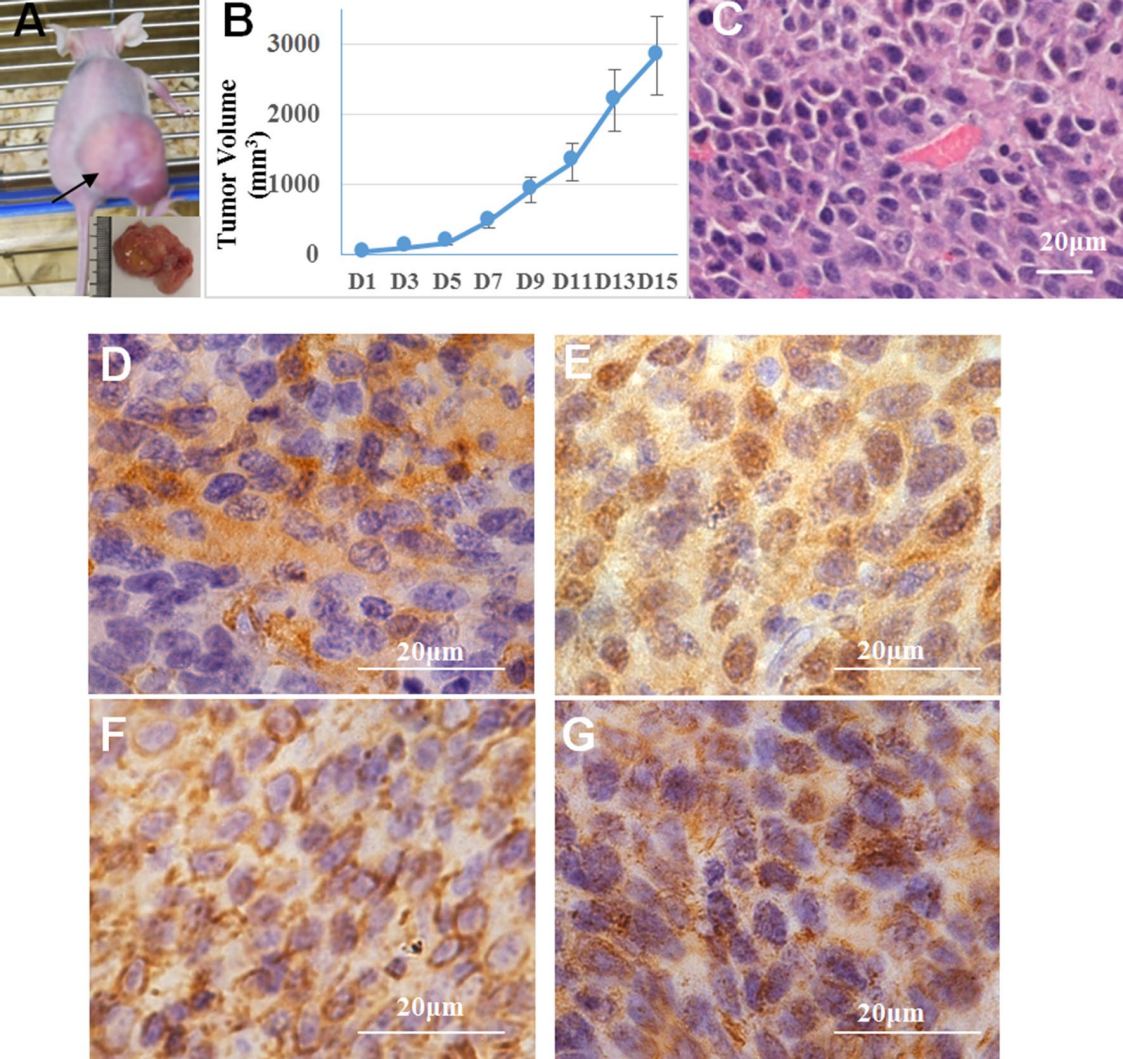
Tumorigenicity of HXWMF-1 cells. **a** Subcutaneous tumor mass in nude mice (arrow indicated). **b** The growth curve of subcutaneous tumors in nude mice (n = 6, age 5–6 weeks). All animal procedures were carried out in accordance with the guidelines established by the internal Institutional Animal Care and Use Committee and Ethics Committee guidelines of Sichuan University. **c** HE staining of the tumor showed that the tumor mass was composed of leukemia cells and blood vessels (×200 magnification). **d**–**f**, **h** On IHC staining, tumors derived from HXWMF-1 expressed α-SMA (**d**), FSP1 (**e**), Vimentin (**f**), and HSP47 (**h**). Original magnification: ×1000

To determine the tumorigenicity of HXWMF-1 cells, we did the colony formation test in vitro, and found that visible colonies appeared on the methylcellulose plate after 6 days, and the clone formation rate was 12.0 ± 4.5%. Then, an in vivo test was conformed; 3 × 10^6^ cells were injected into female BALB/c (nu/nu) nude mice (n = 6) subcutaneously. After 15 days, subcutaneous tumors were observed in 6 mice, with a mean volume of 2835.01 ± 707.98 mm^3^ (n = 3) (Fig. 4a, b). Hematoxylin and eosin (HE) staining indicated that the tumor masses were composed of tumor cells with abundant angiogenesis (Fig. 4c). Immunohistochemistry (IHC) staining showed that α-SMA, FSP1, Vimentin and HSP47 were highly expressed in the xenografts (Fig. 4d–f).

To verify that HXEX-ALL1 can induce the stromal cells malignant transformed, we repeated the above experiments, and obtained another two malignant CAFs cell lines, named HXWMF-2 and HXWMF-3, derived from another two xenografts tumor masses of HXEX-ALL1. The two novel CAFs tumor cells repeated the characteristics of HXWMF-1.

## Discussion

HXWMF-1 is the firstly constructed leukemia associated fibroblastic tumor cell line. To our knowledge, the research first reports that cancer cells can induce the stomal cells to be malignant transfomed, and the HXWMF-1 is the first LAFs/CAFs tumor cell line cultured in vitro.

Among all cells, fibroblasts could be considered the cockroaches of the human body, and survive severe stress that is usually lethal to all other cells, and they are the only normal cell type that can be live-cultured from post-mortem and decaying tissue [14]. They synthesize the extracellular matrix and play a crucial role in maintaining the structural integrity of most tissues. The accurate definition of fibroblasts remains an issue of debate, although they were first defined approximately a century and a half ago as spindle-shaped cells capable of collagen synthesis in connective tissue [15, 24, 27, 28]. For that, fibroblasts are highly heterogeneous in their origins, molecular markers and functions, and they exhibit distinct cellular phenotypes with inherent plasticity and resiliency according to the surrounding microenvironment [14, 24, 28, 29]. Activated fibroblasts were firstly found in wounded healing in 1971[30], and then, in 1979, activated fibroblasts found in TME are termed CAFs [31].

In recent years, extensive research demonstrated that CAFs are the main cellular constituents of the TME in both primary and metastatic cancer [29]. Consistent with fibroblasts, CAFs are highly heterogeneous in their origins, molecular markers and functions also [14, 15, 21, 24, 28, 32]. CAFs may originate from the local microenvironment, or can be recruited to the tumor from a distant source, such as BM [14, 29]. Although the precise origins of CAFs are not fully elucidated, in general, CAFs can be derived from normal resident tissue fibroblasts, fibrocyte and mesenchymal stem cells (MSCs); MSCs and fibrocyte recruited from BM; epithelial or endothelial cells that are adjacent to cancer cells and undergo epithelial- to- mesenchymal transition or endothelial- to- mesenchymal transition; and other cells undergo trans-differentiation, such as adipocyte, pericyte and smooth muscle cells [15]. That is to say, CAFs are not a homogenous cell population, and these cells have diverse functions, implying that tumor-promoting CAF and tumor-suppressing CAFs coexist in the tumor stroma [15, 28, 29, 33].

Targeting CAFs, by altering their numbers, subtype or functionality is being explored as an avenue to improve cancer therapies [28]. However, research in this area faces numerous challenges, not least because CAFs can have both protumorigenic and antitumorigenic effects. The lack of CAFs-specific markers poses a challenge in considering CAFs-target therapy. Therefore, identifying markers of CAFs has become an increasingly fruitful undertaking [14, 24, 27, 28, 32, 34–36]. Owing to their preponderance and inherent plasticity, CAFs are relatively easy to isolate and culture and thus have been extensively studied in vitro.

Recently, we constructed a cell line, HXEX-ALL1, which was originated from a pediatric patient with relapsed B-ALL after contemporary chemotherapy [26]. To explore the microenvironment of relapsed ALL, we tried to construct CAFs cell line from the HXEX-ALL1 xenografts in nude mice. We cannot believe that a LAFs/CAFs tumor cell line was constructed until the cell line was passaged for 50 times and proliferated quickly and stably. Different to HXEX-ALL1, the HXWMF-1 cells grow adhering to the wall with spindle- or polygonal-shape. STR results showed that the cells are originated from mouse. HXWMF-1 cells exhibit all the characteristics of cancer cells [37]: limitless replicative potential with a loss of postconfluence contact inhibition; resisting cell death; self-sufficiency in growth signaling with a colony formation ability in methylcellulose; invasion and metastasis with a rapidly wound healing ability in vitro and a high capacity of tumorigenicity in vivo; genome instability and mutation with a complex karyotype; inducing angiogenesis with an abundant neovasculature in HXWMF-1 xenografts. Interestingly, we got two more fibroblastic tumor cell lines from another two HXEX-ALL1 xenografts with similar characteristics. Using the same method, we did not obtain CAFs tumor cells from xenografts of ALL cell lines, Nalm-6 and CEM-C1-15. That is to say, the phenotype of ALL cells may determinate the phenotype of CAFs. Fortunately, a recent research base on single-cell multiomics sequencing reported that somatic copy number alterations are prevalent in fibroblasts, especially in CAFs, and five genes (*BGN*, *RCN3*,* TAGLN*,* MYL9*, and* TPM2*) are identified as fibroblast-specific biomarkers of poorer prognosis of colorectal cancer [38]. We suppose, under proper microenvironment, the fibroblasts with genetic alteration might be malignant transformed, just as the HXWMF-1 cells; and the TME with malignant TME may bring about the cancer progression and poor prognosis.

CAFs are distinguished from their normal counterparts by the differential expression of markers such as α-SMA, FAP, FSP1, and PDGFR, although none of which was CAFs-specific marker [14, 15, 28, 29]. Recently, increasing evidences showed that CAFs can be classified into myofibroblast phenotype with a high α-SMA expression, and inflammatory phenotype with a low or negative α-SMA expression and secretion of chemokine [28, 35]. In-depth researches showed that CD10^+^GPR77^+^ CAFs may correlate with chemoresistance in breast cancer and lung cancer [34]; PDGFRα negative CAFs may correlate with poor prognosis in breast cancer [36]. Our research showed that α-SMA, FSP1, Vimentin, HSP47, FAP, PDGFRα, PDGFRβ, CD34, LY-6A/E (Sca-1) and CD166 were all positive expressed in HXWMF-1 cells. The exact roles of those markers need to be explored in depth.

For a long time, we found that some patients got a donor cell leukemia (DCL) after bone marrow transplantation [39–44]. DCL was first described in 1971, and for years the incidence of DCL was increasing [39]. The molecular mechanisms for DCL remain unclear. A ‘‘multiple hit’’ hypothesis has been proposed; genetic factors might prime stem cells with a pre-leukemic phenotype within the donor, with a range of recipient- and therapy-specific factors probably interacting to contribute toward realization of malignant potential following engraftment into the more conducive bone marrow environment of the recipient [39–44]. Here, we suppose that the microenvironment with malignant stromal cells may interact with the donor cells and induce the neoplastic transformation. A paper of newly published speculated that certain mutations in the donor cells might have a selective advantage in specific conditions of the BM microenvironment [41]. However, to date there is no firm evidence to support this theory. Fortunately, the novel LAFs/CAFs tumor cell line can help to investigate the mechanisms.

## Conclusions

To the best of our knowledge, HXWMF-1 is the first fibroblastic tumor cell line derived from LAFs or CAFs. In addition, the cell line is the first evidence for that leukemia cells may induce LAFs/CAFs malignant transformation. HXWMF-1 may help to explore in-depth the mechanisms of DCL and BM fibrosis. More important, with this novel cell line, scientists can investigate the molecular mechanisms underlying interaction of TME and ALL cells, and develop brand new theory and therapeutic strategies for patients with ALL, especially for patients with relapsed/refractory ALL.

## Data Availability

The datasets used and analyzed during the current study are available from the corresponding author on a reasonable request.

## Abbreviations

ALL: Acute lymphoblastic leukemia
AML: Acute myeloid leukemia
BM: Bone marrow
CAFs: Cancer-associated fibroblasts
DCL: Donor cell leukemia
LAFs: Leukemia-associated fibroblasts
PDL: Population doubling level
STR: Short tandem repeat
Td: Doubling time
TME: Tumor microenvironment
